# Identification of potentially inappropriate medications with risk of major adverse cardio- and cerebrovascular events among elderly patients

**DOI:** 10.1080/07853890.2021.1897448

**Published:** 2021-09-28

**Authors:** Joana C. F. Lima, João Aguiar, Margarida Paixão Ferreira, Rita Calixto, Vera Cesário, José Vaz

**Affiliations:** a Serviço de Medicina Interna, Hospital José Joaquim Fernandes, Beja, Portugal; b Instituto de Investigação do Medicamento (iMED.ULisboa), Faculdade de Farmácia, Universidade de Lisboa, Lisboa, Portugal

## Abstract

**Introduction:**

Elderly patients pose challenge in clinical practice. Multimorbidity, polypharmacy, and potentially inappropriate medications (PIMs) are a reality among these patients, and can increase the risk of adverse drugs reactions [1]. However, data on the prevalence of PIMs with risk of Major Adverse Cardio- and Cerebrovascular Events (MACCE) in secondary care is scarce. Our aim was to evaluate the prevalence of such PIMs in a Portuguese hospital and to identify the most common pharmacotherapeutic groups involved.

**Materials and methods:**

A cross-sectional study was undertaken in a Portuguese hospital in Beja in the last three months. Patients aged 65 or older with previous cardiovascular disease (considered as ischaemic and haemorrhagic stroke, transient ischaemic attack, and heart failure), and with at least three home medications were included. Data was extracted from medical charts, which included sociodemographic, clinical, and pharmacotherapeutic variables. PIMs with risk of MACCE were identified using a current systematic review. The prevalence of PIMs was defined as the number of elderly patients with medications included in the PIM-list among all the patients included. The informed consent of the subjects and acceptance of the study protocol by a local ethics committee has been obtained. Data analysis was performed using univariate statistics (IBM SPSS v.20.0).

**Results:**

A total of 322 elderly patients were included, where 50.9% (*n* = 164) were female with a mean age of 78.8 ± 10.8 years old. Almost half of the sample presented previous history of cerebrovascular events (47.8%, *n* = 154), followed by heart failure (41.9%, *n* = 135). Patients presented an average of 3.9 ± 2.3 comorbidities per patient and 75.2% (*n* = 242) experienced polypharmacy (defined as the patient taking 5 or more drugs). Each patient was taking a mean of 6.9 ± 3.6 drugs. Thirty nine percent (*n* = 125) of the patients were using PIMs with cardio- and cerebrovascular adverse events, and 23.2% (*n* = 29) of them presented MACCE risk. The most common pharmacotherapeutic group was Non-Steroidal Anti-inflammatory Drugs (NSAIDs) (51.7%, *n* = 15), followed by calcium channel blockers (17.2%, *n* = 5).

**Discussion and conclusions:**

Data suggest that almost 40% of the patients were using PIMs with cardio- and cerebrovascular risk and had previous history of cardiovascular diseases. This may suggest that at discharge and during transitions of care, patients would benefit from a medication review.
